# Longitudinal Growth and Body Composition of Twins versus Singletons in the First Month of Life

**DOI:** 10.1155/2013/108189

**Published:** 2013-12-24

**Authors:** Giulia Paviotti, Angela De Cunto, Laura Travan, Jenny Bua, Gabriele Cont, Sergio Demarini

**Affiliations:** Division of Neonatology, Institute for Maternal and Child Health, IRCCS Burlo Garofolo, Via dell'Istria 65/1, 34137 Trieste, Italy

## Abstract

*Background*. Although twin gestation is well recognized to be associated with impaired fetal and postnatal growth, specific data about body composition of twins in the first month of life are scarce. *Objective*. The aim of this study was to compare the body composition of twins, evaluated with air-displacement plethysmography, to that of singletons of similar gestational age and adequacy of growth, during the first month of life. We tested the hypothesis that the quality of growth would be similar. *Methods*. Anthropometric and air-displacement plethysmography measurements were performed in 18 pairs of twins and in 36 singleton neonates, longitudinally, from birth to the 30th day of life. Each twin was matched to a singleton infant of similar gestational age and birth weight *z*-score. *Results*. With regard to anthropometric measures, the only difference was a lower weight in twins versus singletons on the 15th day of life. With regard to body composition, we did not find any difference between groups at any time point. Fat mass increased significantly from day 1 to day 30 in both twins and singletons. *Conclusion*. In terms of body composition, twins do not differ from singletons of similar gestational age and weight, either at birth or in early postnatal life.

## 1. Introduction

Early nutrition, growth, and subsequent health are closely interrelated [1]. Twins grow less than singletons in utero [2] and postnatally during the first decade of life [3, 4].

Evaluation of body composition (BC) allows assessment of quality of growth in terms of fat mass (FM) and fat-free mass (FFM) and may potentially lead to a better nutritional management in infants with impaired growth [1]. Specific data about BC of twins in early postnatal life are lacking. Air-displacement plethysmography (ADP) is a simple, noninvasive method to measure BC in newborns [5].

The aim of this study was to compare, by ADP, the BC of twins to that of singletons. We tested the hypothesis that quality of growth would be similar.

## 2. Methods

Parents provided written consent; the study was approved by the ethics committee of our institution.

Twins and singletons with a gestational age (GA) between 35 and 40 weeks inborn admitted from 01.2011 to 09.2012 were recruited at birth. Exclusion criteria were intensive care support, oxygen supplementation, intravenous hydration, congenital malformations or chromosomal abnormalities, and having no consent.

We conducted a longitudinal, observational preliminary study. Each twin was matched to a singleton infant of similar GA (±3 gestational days) and birth weight (BW) *z*-score (±0.3).

BW *z*-score was calculated from the BW of infants obtained in the delivery room (Panda Warmer, GE Healthcare, Finland. Accuracy: ±10 g) and BW mean and standard deviation of a Caucasian reference population [6]. Sex was matched whenever possible. GA was based on first trimester sonogram. Data regarding pregnancy were obtained from maternal charts.

Anthropometric measures were taken at birth and on days of life 15 and 30. Recumbent length was measured to the nearest 0.2 mm (Seca gmbh and co. Kg, Hamburg, Germany). Head circumference (HC) was measured as occipitofrontal circumference to the nearest 1 mm with a graduated, nonstretchable, flexible tape measure. FM and FFM were measured by an ADP system (PeaPod Infant Body Composition System, Life Measurement Inc., Concord, CA) in all subjects at three time points: during the first 24 hours of life and on days of life (DOL) 15 and 30. A detailed description of the PeaPod operating principles, validation, and measurement procedures is provided elsewhere [5].

Infants >2500 g at birth were managed within a rooming-in program. They were exclusively breastfed, provided their weight loss did not exceed 10% of BW. All babies <2500 g at birth received 80 mL/Kg/day of breast or formula milk. Feeding was recorded at every measurement as exclusive breastfeeding, partial or predominant breastfeeding, and formula feeding (WHO definitions).

Variables were expressed as mean (standard deviation) as normally distributed. A Student's *t*-test for paired samples was used to compare data of twins versus singletons; a chi-square test or Fisher's test was used to compare proportions. Repeated measurements were compared with a one-way analysis of variance (ANOVA). The association between two continuous variables and the independent effect of multiple variables on a dependent continuous variable were tested with linear and multiple regression analysis, respectively. A cluster correction included in the multiple regression analysis accounted for the matching of data. Statistical significance was assumed at *P* < 0.05. Statistical analyses were performed using MedCalc software rel. 9.3.9.0 (MariaKerke, Belgium) and STATA (StataCorp LP, TX, US).

A total sample size of 68 infants was required to detect a 2% difference in the percent FM, with a power of the study of 80% and an alpha error of 0.05 (equation for paired studies).

## 3. Results

Table 1 summarizes the clinical and demographic data of the 36 twins and of the 36 matched singleton controls, together with their anthropometric and BC data, on DOL 1, 15, and 30. Pregnancies and perinatal periods were uncomplicated. All infants were Caucasian. Prepregnancy maternal body mass index (BMI) of twins did not differ from that of singletons (21.4 (3.2) versus 22.3 (5.1)), whereas weight increase during pregnancy was higher in the former group (15.9 (5.6) Kg versus 12.4 (4.4), *P* = 0.05). Dizygotic twin couples were 16/18.

Weight measured on day 1 by ADP was lower than BW obtained in the delivery room in both groups (*P* < 0.001). Day 1 BC measurements were performed at 21.6 (9.5) hours of life in twins and at 22.0 (9) hours of life in controls (*P* = 0.9).

No difference in growth or in % FM between twins and singletons was detected at any study point, apart from a lower weight of twins versus singletons at DOL 15, which did not result in a different BC. FM significantly increased from day 1 to day 30 in both groups (*P* < 0.001).

In a multiple regression analysis model (including GA, BW *z*-score, sex, maternal prepregnancy BMI, and weight increase during pregnancy), the % FM on DOL 1 was not significantly associated with twin rather than singleton status. Female sex was weakly associated with a higher %FM on day 1 (*P* = 0.05).

A higher % FM on DOL 1, a lower BW *z*-score, and exclusive breastfeeding were independently associated with a higher % FM on DOL 30 in multiple regression analysis (Table 1).

## 4. Discussion

Twin gestation is associated with impaired fetal and postnatal growth [2–4], but there are limited data about BC of twins. To our knowledge, this is the first study to report longitudinal data on BC of twins versus singletons in early life. Our results show that the intrauterine growth of twins does not result in a different content in FM or FFM at birth, when compared to singletons of similar GA and adequacy of growth. Additionally, we found no difference in % FM between twins and singletons, in the first month of life.

BC of twins at birth was previously assessed [7, 8] using dual energy X-ray absorptiometry (DEXA). Demarini et al. found similar BC for normally grown twins and singletons [7]. Percent FM values were higher than those we reported, probably due to DEXA employment.

In our study, % FM on day 1 was lower than birth % FM of term infants, measured by ADP [9]. Such result is expected, given the shorter GA and lower BW of our infants. Indeed, FM accretion takes place mainly in the third trimester of pregnancy and FM correlates directly with BW [1].

BC of twins was similar to that of singletons also on DOL 15 and DOL 30. This result seems to be in contrast with the reported reduced postnatal growth of twins [3, 4]. However, these data generally lack a control group of singleton infants growth-restricted at birth. Instead, twins who are small at birth account predominantly for the poor postnatal growth of the whole group of twins [3].

We used a multiple regression analysis model to account for the differences in feeding between groups, which may influence BC [10]. Eventually, the results did not change.

In both twins and singletons, % FM showed an approximately threefold increase from birth to day 30. This is in contrast with the twofold increase in % FM, reported in term infants, over the same time period [9]. Feeding is an unlikely explanation for this result as breastfeeding, reportedly associated with a higher postnatal % FM [10], was less common in our group. Rather, slight population differences may be involved. Our newborns were late preterm/early term, moderately growth restricted infants: both conditions have been associated with a rapid postnatal FM increase [11]. The inverse relationship between BW *z*-score and % FM at one month is consistent with this finding.

This is a preliminary report with several limitations. (1) The sample size is relatively small. However, it can detect a 2% difference in % FM and matching by *z*-score, within a narrow range of BW and GA, allows a valid comparison of BC between twins and singletons. (2) The follow-up is short and might fail to show long-term effects of postnatal factors (i.e., feeding) on BC. (3) We could not evaluate the effect of zygosity, umbilical cord insertion, or fusion of placentas on BC [2]. (4) Feeding was evaluated only qualitatively, according to the type of milk. All infants were fed ad libitum; therefore, quantitative data were difficult to measure. However, the variable “exclusive breastfeeding” was accounted for in multiple regression analysis. (5) Matching for sex was not always feasible and the mothers of twins gained more weight during pregnancy. However, the multiple regression analysis accounted for both.

In conclusion, BC of twins does not differ from the one of singletons of similar GA and adequacy of growth, both at birth and in early postnatal life. A rapid FM gain occurs in the first month of life in this population. Our findings need to be confirmed in larger cohorts of twins.

## Figures and Tables

**Table 1:** Growth and body composition of twins versus matched singletons.

	Twins (*n* = 36)	Singletons (*n* = 36)
Gestational age at birth (weeks)	36.9 (1.5)	37.1 (1.4)
Birth weight (g)	2440 (360)	2450 (370)
Birth weight *z*-score	−1.30 (0.66)	−1.32 (0.77)
Boys/girls	23/13	23/13
DOL 1		
BM (g)	2360 (360)	2380 (350)
% FM	5.85 (3.41)	4.97 (2.73)
FM (g)	140 (90)	120 (70)
FFM (g)	2230 (330)	2270 (320)
Length (cm)	46.6 (1.9)	45.9 (2.2)
HC (cm)	32.3 (0.9)	32.2 (1.2)
DOL 15		
BM (g)	2690 (350)*	2850 (400)*
% FM	9.74 (3.92)	10.01 (4.56)
FM (g)	270 (120)	290 (160)
FFM (g)	2430 (290)	2510 (320)
Length (cm)	48.6 (2.6)	48.7 (3.1)
HC (cm)	33.9 (0.9)	34.0 (1.6)
EBF	7*	22*
PBF	22*	7*
EF	7	5
DOL 30		
BM (g)	3400 (380)	3460 (490)
% FM	15.86 (3.64)	16.74 (4.28)
FM (g)	540 (140)	590 (200)
FFM (g)	2860 (340)	2870 (370)
Length (cm)	51.0 (2.0)	51.3 (2.5)
HC (cm)	35.7 (1.0)	35.3 (1.6)
EBF	3*	26*
PBF	24*	6*
EF	9	4

Data are reported as mean (SD) or number of infants. BM: body mass. FM: fat mass. FFM: fat-free mass. HC: head circumference. DOL: day of life. EBF: exclusive breastfeeding. PBF: partial or predominant breastfeeding. EF: exclusive formula feeding. *P < 0.05.
